# Molecular Mechanisms Underlying the Cardiovascular Toxicity of Specific Uremic Solutes

**DOI:** 10.3390/cells9092024

**Published:** 2020-09-02

**Authors:** Jonathan D. Ravid, Vipul C. Chitalia

**Affiliations:** 1 Renal Section, Department of Medicine, Boston University School of Medicine, Boston, MA 02118, USA; jravid@bu.edu; 2 Boston Veterans Affairs Healthcare System, Boston, MA 02130, USA; 3 Global Cocreation Labs, Institute of Medical Engineering and Science, Massachusetts Institute of Technology, Cambridge, MA 02139, USA

**Keywords:** uremic toxins, chronic kidney disease, cardiovascular disease

## Abstract

Mounting evidence strongly suggests a causal link between chronic kidney disease (CKD) and cardiovascular disease (CVD). Compared with non-CKD patients, patients with CKD suffer disproportionately from CVD and derive suboptimal benefits from interventions targeting conventional CVD risk factors. Uremic toxins (UTs), whose plasma levels rapidly rise as CKD progresses, represent a unique risk factor in CKD, which has protean manifestations on CVD. Among the known UTs, tryptophan metabolites and trimethylamine N-oxide are well-established cardiovascular toxins. Their molecular mechanisms of effect warrant special consideration to draw translational value. This review surveys current knowledge on the effects of specific UTs on different pathways and cell functions that influence the integrity of cardiovascular health, with implication for CVD progression. The effect of UTs on cardiovascular health is an example of a paradigm in which a cascade of molecular and metabolic events induced by pathology in one organ in turn induces dysfunction in another organ. Deciphering the molecular mechanisms underlying such cross-organ pathologies will help uncover therapeutic targets to improve the management of CVD in patients with CKD.

## 1. Introduction

### 1.1. Overview

The prevalence of CKD might be approaching pandemic levels in the United States, with about 30 million people suffering from the disease, and with significant associated rates of morbidity and mortality [1]. According to the Center for Disease Control, 15% of US adults are predicted to develop CKD in their lifetime. Morbidity and mortality in CKD patients are largely related to CVD [2]. This includes heart failure, atherothrombotic disorders driving myocardial infarction and stroke, and arrhythmias and sudden cardiac death in adult and pediatric populations [3]. Given the profound public health implications of CVD in CKD, it is imperative to target CKD for primary and secondary prevention of cardiovascular complications. For this translation to the human realm, an in-depth understanding of the mechanisms underlying the toxicity of certain UTs is needed.

### 1.2. Uremic Solutes

A uremic milieu is characterized by retention of a host of chemical compounds, which results in injury to various organs. These compounds are termed uremic solutes or uremic toxins. Of several varieties of UTs, protein bound uremic toxins (PBUTs) are particularly toxic to the cardiovascular system [4,5,6]. Specifically, indoxyl sulfate (IS) [7,8], p-cresol/p-cresyl sulfate (PCS) [9,10], trimethylamine N-oxide (TMAO) [11], kynurenine (Kyn) [12], and indole-3-acetic acid (IAA) [13] have been well characterized for their cardiovascular toxicity. These uremic solutes are generated by mammalian processing of metabolites of gut microbial catabolism, and are excreted by renal tubular secretion [14,15]. The source of PBUTs includes tryptophan metabolites such as IS, Kyn, and kynurenic acid (KA). After absorption, the majority of tryptophan is metabolized to bioactive molecules through the activities of tryptophan dioxygenase (TDO) or hepatic indoleamine dioxygenase (IDO) to form Kyn, which is then further catabolized to compounds, such as kynurenic acid and quinolinic acid, as reviewed in [16]. The remaining tryptophan is metabolized by intestinal microbes expressing tryptophanase, an enzyme which generates indols. Indols, in turn, are converted to IS by hepatic cytochrome P450 family 2 subfamily E member 1 (CYP2E1) and then microsomal sulfotransferase family A1 member 1 (SULT1A1). While these PBUTs are the products of amino acids, uremic toxins like trimethylamine N-oxide (TMAO) are derived from a combination of choline, L-carnitine, and phosphatidylcholine [17,18]. The plasma level of TMAO can be elevated after ingestion through a fish diet, or can originate from liver metabolism of trimethylamine (TMA), a gut bacteria product, to TMAO [19]. A five-year follow-up study of 521 CKD patients found that a higher level of plasma TMAO is a predictor of shorter survival, and, in accordance, chronically elevated TMAO levels in rats induced renal fibrosis and renal pathology [18].

**Figure 1:**
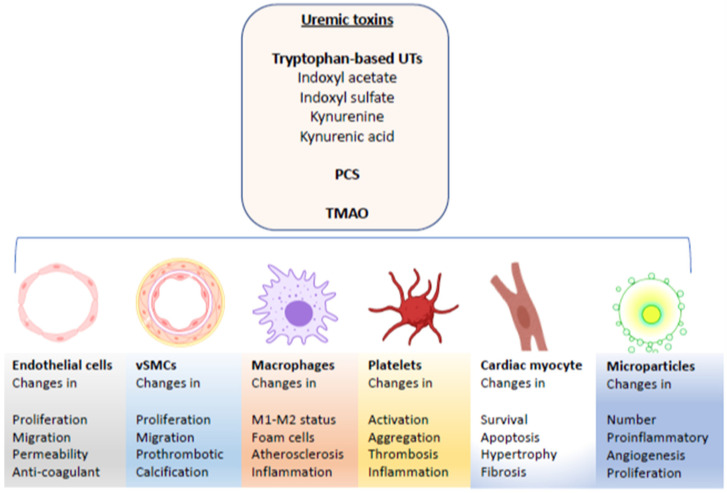
Pleotropic effects of uremic toxins on various cells involved in CKD-associated cardiovascular disease.

In this review, we will focus on the effects of a set of UTs on various cell types and organs within the cardiovascular system, as well as survey mechanisms by which specific molecules impact such cells. UTs contribute to several cardiovascular pathologies, including accelerated atherosclerosis and neointimal hyperplasia, hyperthrombotic state, abnormal vascular calcification, and microvasculature rarefaction and suppressed angiogenesis (Figure 1) [20,21,22,23,24,25,26,27,28]. Because these pathological processes involve a large array of cells, the following account summarizes the effect of a set of UTs (tryptophan metabolites and TMAO) on various cell types, and how these specific perturbations are integral to CVD pathogenesis in CKD patients.

## 2. Effects of UTs on Endothelial and Endothelial Progenitor Cells

The vasculature is the largest organ system in the body, and UTs come in contact first with endothelial cells. UTs have been linked to endothelial dysfunction, which is observed starting even in the early stages of CKD, and is almost universal in later stage CKD patients [29]. Endothelial cell (EC) dysfunction manifests with a myriad of pathophysiological disturbances compromising EC survival, migration, autophagy, and alterations in the permeability and coagulation properties of the vasculature.

### 2.1. Alteration in Mitogenic Signaling in Endothelial Cells and Endothelial Progenitor Cells

EC proliferation, migration and survival are critical for several biological processes such as angiogenesis and recovery after vascular injury characterized by an endothelial wound. Indolic solutes at plasma levels seen in CKD suppressed EC survival and migration, as well as chemotactic motility of endothelial cell progenitors (EPCs) [30,31,32]. The mitogen-induced pro-proliferation and pro-survival signaling pathways are important for EC proliferation and survival. PBUTs modulate different pathways to suppress fundamental EC functions. The mitogen-activated protein kinase (MAPK) pathway is a well-characterized mitogenic pathway, known to regulate the cell cycle, cell survival and proliferation through various mechanisms. Proteins in this pathway are members of a family of serine/threonine kinases, and are traditionally grouped into three main sets: extracellular signal-regulated kinases (ERK), C-Jun N-terminal kinases (JNK), and p38 MAPKs [33]. Similar to any mitogenic pathway, the MAPK signaling pathway is initiated with the engagement of the ligand with the receptor, which, in turn, triggers a signaling cascade in the cells, resulting in cellular response. The MAPK signaling consists of a cascade of kinases that propagate the activation of kinases through phosphorylation status of pathway members. Buendia et al. treated human umbilical vein endothelial cells with sera obtained from CKD patients and observed hyperphosphorylation of ERK1/2 in these cells as compared to cells treated with normal serum [34]. The MAPK pathway is activated within 30 min of treatment with IS, as reflected by phosphorylation of downstream signaling molecules, such as p38 and ERK1/2 [34]. Taken together, these studies in different systems collectively point to the MAPK pathway as a mediator of the effect of UTs on vascular cells.

**Figure 2:**
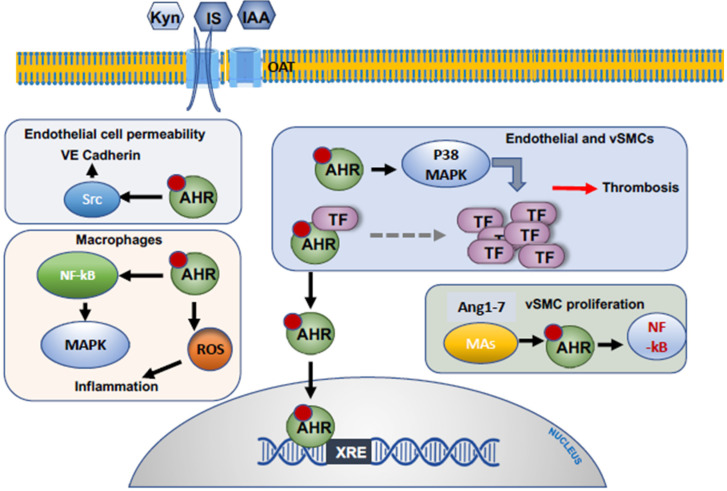
Examples of molecular mechanisms underlying the effects of uremic toxins on various cells, with focus on the aryl hydrocarbon receptor signaling (AHR) pathway. Indolic uremic solutes and kynurenine, all tryptophan metabolites, are agonists of the AHR pathway. These solutes enter cells through the organic anion transport channel (OAT) in target cells. Upon binding to the cytosolic AHR protein, the ligand-bound AHR (represented as a red circle on AHR) elicits distinct signaling cascades in various cell types. In endothelial cells, AHR activation regulates cell permeability through Src and prothrombotic propensity through tissue factor (TF) activation or through p38 mitogen-activated protein kinase (MAPK) signaling. AHR activation in vSMCs induces thrombosis through TF stabilization and by activating the NF-κB pathway. In macrophages, AHR induces a proinflammatory state by increasing reactive oxygen species, and through the NF-κB-MAPK signaling cascade.

The MAPK signaling pathway also experiences cross-talk with other signaling events. It is known that ERK1/2 (a component of the MAPK pathway) regulates components of the NF-κB pathway, namely p65 a/p50. The nuclear translocation of p65/p50 signifies the activation of the NF-κB pathway, and regulates the expression of several chemokines, such as of monocyte chemoattractant protein-1 (MCP-1). This event may suggest a cross-talk between the mitogenic pathway and the pro-inflammatory signaling regulated by the NF-κB pathway. This model is supported by the observation that treatment of cells with specific inhibitors of ERK1/2 and p38 MAPK suppressed p65 phosphorylation and induction of MCP-1 induced by IS [34]. It is thus possible that IS activates the MAPK pathway indirectly. Indolic solutes (IS and indoxyl acetate) are known ligands for the aryl hydrocarbon receptor signaling (AHR) pathway. Indoxyl acetate or indolic acetic acid (IAA) binds to AHR and activates the AHR pathway (more details in the following section). Addi et al. noted that AHR signaling regulates tissue factor (TF), a procoagulant protein highly expressed in vascular cell types such as endothelial cells, vascular smooth muscle cells, and pericytes. Stimulation of AHR induced TF through activation of p38 MAPK and binding to NF-κB to the TF promoter. The later event was mitigated by specific inhibitors of p38 MAPK [35] (Figure 2). Taken together, the above studies showed stimulation of mitogenic pathways with the treatment of cells with PBUTs to regulate various cellular functions in the uremic milieu.

Uremic solutes also affect EPCs, which together with ECs are critical in angiogenesis. In this process, along with ECs, EPCs are recruited to form angiogenic buds for new vessel formation. IS diminishes hypoxia-induced EPC migration and capillary tube formation [36]. Hung et al. showed that mice that underwent subtotal nephrectomy had significantly increased plasma levels of IS, and had reduced reperfusion and impaired neovascularization in ischemic hindlimbs compared with control mice that underwent sham operation. Increased IS levels were also associated with diminished levels of phospho-eNOS, phospho-signal transducer and activation of transcription (STAT)3, and vascular endothelial growth factor (VEGF). The collective phenotype was reversed by treating the mice with an oral adsorbent of UTs, AST-120. Concordant cell culture studies using human EPCs showed a suppressive effect of IS on VEGF expression through interleukin-10/STAT3 signaling [36].

### 2.2. Alteration of the Procoagulant Function of Endothelial Cells

EC proliferation is critical for the rapid reendothelialization that is warranted during repair of endothelial wounds inflicted spontaneously during rupture of an unstable plaque, or caused by endovascular procedures. Poor endothelial wound repair is likely to expose the highly prothrombotic subendothelial layer to form a reactive vascular bed for thrombogenesis. In general, healthy endothelium is considered anti-coagulant, preventing aberrant activation of the coagulation cascade and fibrin formation and/or platelet activation. Delayed reendothelialization secondary to decreased endothelial cell proliferation and migration is related to increased risk for delayed thrombosis observed after endovascular therapy in CKD patients [37,38]. This is especially relevant to CKD patients, as adequate vascular access sites for hemodialysis (HD) often serve as their lifeline, and these sites are especially susceptible to thrombosis in the setting of repeated endothelial injury [39,40,41].

Healthy endothelial monolayers exhibit anti-inflammatory and anti-coagulant properties [42]. Endothelial injury or endothelial dysfunction activates the extrinsic coagulation system, in which TF serves as a primary trigger for fibrin generation, creating a nidus for thrombus formation [43,44]. Indolic solutes such as IS or IAA activate the aryl hydrocarbon receptor (AHR) pathway in ECs to upregulate TF [45]. AHR is a member of the family of basic helix–loop–helix transcription factors, which was first identified over three decades ago, and described then as a regulator of metabolism of xenobiotics and toxicity [46]. Since then, various ligands have been found to activate AHR. The cytosolic non-activated form of this receptor appears as a complex with several chaperone proteins such heat shock protein 90 and a co-chaperone p23 [47]. In this folded configuration, AHR recognizes its ligand to allow nuclear localization. In the nucleus, it binds to the promoter of its target genes [48]. AHR upregulates TF mRNA to increases its surface expression [39] (Figure 2). IS and IAA binding and activation of AHR also result in increased release of microparticles loaded with TF. In support of this, the blood levels of IAA, IS, and PCS in CKD patients are associated with a reduced number of circulating endothelial progenitor cells, signs of vascular injury, and increased plasma levels of endothelial microparticles (EMPs) [39]. Addi et al. examined a wide array of endothelial cells such as human umbilical veins, human aortic endothelial cells, and cardiac-derived microvascular cells [35]. They observed that treatment of cells with IAA resulted in AHR activation and TF upregulation. However, AHR was not found to bind to the TF gene promoter, suggesting a possibility of an alternative mechanism or cross-talk among signaling pathways. Interestingly, their analysis of the TF promoter showed that NF-κB was essential in TF induction by IAA. In support of these findings, an inhibitor of the NF-κB pathway reduced the effect IAA on TF (Figure 2). Taken together, these studies showed that different UTs regulate TF and thrombosis through various pathways and cross-talks.

ECs are also responsible for the production of prostaglandins, which are important in hemostasis. In detail, ECs produce prostaglandin E2 (PGE2) from arachidonic acid via cyclooxygenase 2 (COX-2). In turn, PGE2 has been shown to upregulate platelet aggregation and lead to hypercoagulable state through binding to the platelet EP3 receptor. In a study of cultured human ECs, IAA led to COX-2 upregulation via an AHR/p38 MAPK/ NF-κB pathway, leading to increased endothelial production of PGE2, suggesting a potential mechanism of the hypercoagulable state in uremia.

### 2.3. Inflammation, Oxidative Stress and Atherosclerosis

UTs are pro-inflammatory, which is also known to activate ECs to expresses TF, thereby augmenting coagulation and thrombosis [49,50]. Inflammation is the fundamental driver of atherosclerosis. Of all UTs, tryptophan metabolites such as IS, IAA, and indoxyl-β-d-glucuronide and p-cresyl sulfate (pCS) are especially implicated in inflammation [13,51]. In specific, CKD patients stages 3–5 followed for 5.2 years showed a strong correlation between the level of IS, IAA, and pCS and various markers of vascular inflammation, such as IL-6, C-reactive protein (CRP), monocyte chemoattractant protein-1 (MCP-1), soluble vascular adhesion molecule-1 (sVCAM-1), and soluble intercellular adhesion molecule-1 (sICAM-1) [52]. A correlation between IAA levels and CRP in CKD patients was also confirmed in another report [13]. In a cohort of 2399 CKD patients followed for 7.3 years as well as in a cohort of HD patients, increased inflammation and IS levels were independently associated with atherosclerotic vascular disease [53,54].

The direct effect of uremic toxins on inflammatory molecules and oxidative stress was evaluated in different EC cultures. A human EC line treated with normal or uremic sera from end-stage renal disease (ESRD) patients with diabetes and/or hypertension showed an upregulated expression of known inflammation markers such as MCP-1 and stromal cell-derived factor 1 (SDF-1), as compared to cells incubated with healthy sera [55]. The role of AHR in regulating some of these inflammatory processes was established using mice in which AHR was specifically knocked out in endothelial cells [56]. IS treatment induced expression of E-selectin on ECs and increased adhesion of inflammatory leukocytes to the endothelium. EC inflammation and adhesion of leukocytes are key processes in the progress of atherosclerosis. This effect was diminished in AHR knockout mice as compared to controls [56]. In accordance, other studies showed that activation of AHR by dioxins induces macrophage-mediated inflammation, appearance of cholesterol laden foam cells, and increased atherosclerotic lesions in ApoE deleted mice [57].

IAA upregulates the AHR/p38 MAPK/NF-*κ*B pathway, and augments the generation of reactive oxygen species (ROS) in endothelial cells [13] (Figure 2). pCS and IAA incubated with human ECs each inhibit autophagic processes and cause accumulation of carbonylated proteins, again indicative of oxidative stress [58]. Further, in human umbilical vein ECs, IS increased the level of reactive oxygen species generated by the cells, as well as mitochondrial depolarization, while decreasing mitochondrial mass and function [59]. In the same cell system, IS also induced senescence by increasing the level of reactive oxygen species (ROS) [60]. Oxidative stress is also linked to endothelial dysfunction characterized by derangement in EC-dependent flow-mediated vessel relaxation. In a study using human umbilical vein endothelial cells (HUVECs), IS upregulated expression of NADPH oxidases (Nox4, Nox2) and production of ROS, with consequent downregulation of nitric oxide (NO) expression, the primary mediator of this phenomenon [61]. Accordingly, acetylcholine-induced endothelium-dependent relaxation of a rat superior mesenteric artery was reduced by IS, owing to its negative effect on NO production [62]. In conclusion, the above studies strongly suggest the ability of protein bound uremic solutes to induce pro-inflammatory and oxidative stress within a variety of cell types augmenting atherosclerotic cardiovascular disease [56,57,60].

### 2.4. Endothelial Cells and the Permeability Barrier

The endothelial cell layer regulates permeability using two distinct pathways—transcellular and paracellular pathways to tightly modulate the movement of circulating cells from the blood into the vessel walls, and then into the interstitium. This layer also regulates the movement of nutrients and electrolytes. Alterations in the endothelial barrier are reflected by disrupted endothelial morphology and reduced expression of the cell-to-cell junction proteins vascular endothelial (VE)-cadherin and zonula occludens-1 in the endothelium. Studies have shown augmented endothelial permeability in the aorta of experimental models of CKD [63,64]. In cultured ECs, both VE-cadherin and ZO-1 protein expression decreased after exposure to uremic serum [65]. VE-cadherin mRNA expression was reduced after exposure to PCS, IS, and uremic serum. Assefa et al. exposed bovine aorta endothelial cells to IS, subsequently noting increased cell permeability [63]. As noted earlier, in this case too, IS treatment resulted in the activation of AHR signaling as well as Src, a non-receptor tyrosine kinase linked to remodeling of actin cytokine machinery. The AHR inhibitors CH223191 and resveratrol, a dietary polyphenol, inhibited IS-induced enhanced cell permeability. Similarly, treatment with a Src inhibitor abolished Src activation and endothelial cell permeability (Figure 2). These results support an IS-AHR/Src/VE-cadherin axis regulating endothelial hyperpermeability. While other factors such as vitamin D deficiency in CKD patients are also shown to disrupt VE-cadherin interactions and F-actin reorganization [64], the direct effect of UTs on critical components of endothelial permeability is illustrated by the above studies. Irrespective of mechanism, a compromised endothelial barrier affects the traffic of molecules and solutes between the vessel lumen and the vessel wall, and these processes are mechanistically related to the development of atherosclerosis [66].

## 3. Effects of UTs on Vascular Smooth Muscle Cells

### 3.1. Effects of Uremic Toxins on Mitogenic Signaling in Vascular Smooth Muscle Cells

Derangement of vascular smooth muscle cells (vSMCs) is a quintessential component of uremic vascular disease. vSMC proliferation and hyperplasia are hallmarks of atherosclerosis and intimal stenosis, and constitute a major contributor to thrombosis in arterial diseases. Several UTs such as inorganic phosphate or IS have been shown to induce vSMC proliferation, migration, and calcification [67], in part through activation of the MAPK pathway [68]. Using both CKD rats and IS-administrated rats, Yisireyili et al. showed that IS activated prorenin receptors in vSMCs through oxidative stress, AHR stimulation, and NF-κB activation [69]. Administration of N-acetylcysteine (an antioxidant) and diphenyleneiodonium (an inhibitor of nicotinamide adenine dinucleotide phosphate oxidase), or knockdown of AHR and NF-κB, inhibited IS-induced expression of prorenin in vSMCs [69]. Moreover, silencing of prorenin abrogated IS-induced vSMCS proliferation and TF expression. Other mediators of IS-induced vSMC proliferation include reactive oxygen species, mitogenic pathways such as platelet-derived growth factor-ß (PDGFβ), and regulation of cell cycle proteins such as cyclin D1 and p21, p53 and Glut1. Muteliefu et al. showed that IS significantly promoted the proliferation of human aortic vSMCs in a concentration dependent manner, but which was significantly suppressed by antioxidants such as vitamin E, vitamin C, and N-acetylcysteine [70]. PDGF is a mitogenic ligand that binds to PDGF receptor (PDGFR), a tyrosine kinase receptor pathway receptor, to activate downstream signaling. IS increased the sensitivity of vSMCs to PDGF and augmented their proliferation [71]. PDGF stimulation also increased reactive oxygen species production, thereby upregulating vSMC proliferation through this mechanism. The effect of IS was specifically observed through the PDGFβ receptor. In addition, IS-induced vSMC proliferation was suppressed by inhibitors of ERK and p38 MAPK pathways [71].

IS also exploits pathways mediated through the stimulation of the MAS1 receptor, a G protein-coupled receptor which binds the angiotensin II (Ang II) metabolite angiotensin-(1-7) (Ang-(1-7)) [72]. Ang-(1-7), by acting via the Mas receptor, exerts inhibitory effects on Ang-II and suppresses inflammation and vascular and cellular growth mechanisms [73]. IS downregulated the Mas receptor in the aorta of normotensive and hypertensive rats [72]. This effect was mitigated by silencing of AHR and NF-κB. Ang (1-7) attenuated IS-induced cell proliferation and TF expression in vSMCs, and exerted this effect through by inhibiting mitogenic signaling cascades such as ERK1/2 and NF-κB [72].

IS also regulates vSMCs proliferation through the induction of glucose transporter-1 (GLUT1) expression [74]. Lin et al. noted that GLUT1 facilitates the transport of glucose into vSMCs, and GLUT1 overexpression increases vSMC proliferation [74]. IS induced a significant increase in expression of GLUT1 protein as well as of pro-proliferative cyclin D1 and p21 mRNA, in addition to a modest increase in expression of antiapoptotic p53 mRNA in vSMCs. In this system, IS also significantly suppressed Akt phosphorylation after 6 h and 12 h treatments of vSMCs, and increased S6K phosphorylation (involved in protein translation) after a 3 h treatment. Treatment of vSMCs with rapamycin, a well-established inhibitor of mTOR signaling, mitigated the effect of IS on GLUT1 expression and vSMC proliferation [74].

In addition to the generation of atherosclerotic plaque, vSMC proliferation is central to vascular remodeling following injury. Treatment of ECs or vSMCs with pCS at concentrations typically found in CKD patients induced oxidative stress in them, and incubation of aortas with pCS for two days caused vascular remodeling characterized by luminal narrowing [75]. A study involving atherosclerosis prone ApoE(-/-) mice showed that 5/6 nephrectomy followed by eight weeks of pCS administration resulted in greater development of atherosclerosis and vSMC proliferation as compared with control mice [76].

### 3.2. Effect of Uremic Toxins on Neointimal Hyperplasia and Calcification

Neointimal hyperplasia (NH) is characterized by increased number of vSMCs and deposition of proteoglycans in the intima. NH is enhanced in the uremic milieu [77], and is frequently observed in context of arteriovenous fistulae (AVFs) [78]. Using a murine model of CKD, the mechanism leading to NH was probed after AV fistula surgery. An increase in NH at the fistula site in CKD animals as compared with controls was not associated with increased vSMC proliferation, but rather augmented cell migration, as examined in aortic explants from CKD mice. Interestingly, a uremic milieu induced expression of osteopontin in vSMCs, suggesting their programming to osteoblasts. Accordingly, enhancing vSMC differentiation with bone morphogenic protein-7 (BMP7) prior to AV anastomosis prevented the development of NH [77]. Taken together, the above studies indicate that a uremic milieu and IS induce vSMC proliferation as well as migration through a host of mechanisms, in turn promoting the development of pathologies such as NH and atherosclerosis.

Calcification of the vascular media layers is another common complication in CKD patients owing to vSMCs shifting to osteoblastic programming. Cultured primary human umbilical vein smooth muscle cells exposed to different concentrations of IS increased calcium deposition and augmented the expression of osteoblast-specific proteins [79,80]. Several pathways have been studied to deconvolute the mechanisms underlying the effects of IS on vSMC calcification. One study reported an effect through upregulation of 1α-hydroxylase, which is responsible for hydroxylation of calcifediol to calcitriol (the bioactive form of Vitamin D) [81], and other studies have suggested a role of the PI3K/Akt/NK-κB axis [82] or regulation of Wnt/β-catenin signaling mediated through microRN-29b [83]. Zhang et al. demonstrated that vascular miR-29b was down-regulated in radial arteries of patients with end-stage renal disease (ESRD). IS also decreased miR-29b expression in human aortic vSMCs and potentiated their calcification [83]. They observed increased expression of Wnt7b/β-catenin in radial arteries of ESRD compared with a control group. IS increased Wnt7b/β-catenin expression in vSMCS within three days of exposure. They further demonstrated that miR-29b indeed downregulated Wnt7b/β-catenin signaling. These results showed that IS induces expression of miR-29b, in turn suppressing Wnt//β-catenin signaling to induce vascular calcification in CKD a milieu. Similar to IS, pCS augmented osteoblastic markers such as alkaline phosphatase and osteopontin in human aortic vSMCs, partially mediated through pCS-induced expression of NOX4 [84].

### 3.3. Prothrombotic Effects of Specific Uremic Toxins

An important mediator of vSMC-driven cardiovascular pathologies is TF, as it controls several processes [50]. The circulating levels of TF in CKD patients positively correlate with plasma levels of IS, IAA [45], and Kyn, another tryptophan-based uremic solute [85]. IS level is also positively correlated with increased levels of von-Willebrand factor (vWF). As noted earlier, TF is a cell-surface protein; the dysregulation of which contributes to the development of thrombotic events. Our studies showed increased TF activity on the surfaces of primary human aortic vSMCs and endothelial cells in response to sera from CKD patients with varying stages of disease [86,87,88,89]. Human vSMCs pretreated with uremic serum obtained from ESRD patients, or with indolic solutes, and subsequently exposed to a coronary-like blood flow system, demonstrated a significantly greater clot formation. This was in turn reduced with an anti-TF neutralizing antibody [88]. Further mechanistic probing revealed that IS inhibited TF protein ubiquitination and subsequent proteasomal degradation [86,90]. As proposed earlier [91], recent studies showed that IS-induced TF upregulation leading to thrombosis is mediated through AHR signaling [88]. Upon activation by IS, the cytosolic AHR bound to TF and protected it from ubiquitination and proteasomal degradation by an E3 ubiquitin ligase STIP1 homology and U-box-containing E3 ubiquitin ligase protein 1 (STUB1). AHR inhibitor reduced TF-AHR interaction and augmented TF ubiquitination by STUB1 in vSMCs. In fact, a STUB1 activator, YL-109, augmented TF degradation. Both AHR inhibitor and STUB-1 activator significantly mitigated IS and uremic serum-mediated thrombosis in flow loops coated with vSMCs, in a FeCl_3_-induced carotid artery injury model in the setting of CKD, and in an IS-specific solute model [88]. Importantly, in this study, AHR inhibitor and STUB-1 activator ameliorated the effects of IS on a thrombotic phenotype without affecting bleeding time, suggesting that targeting CKD-specific mechanisms can result in safer antithrombotics [88]. In accordance with these molecular studies, analysis of sera from a CKD cohort from the Dialysis Access Consortium Clopidogrel Prevention of Early AV Fistula Thrombosis trial or the Thrombolysis in Myocardial Infarction II trial showed that patients with subsequent arteriovenous thrombosis had significantly higher serum levels of IS and Kyn, and they induced greater AHR and TF activity in endothelial cells and vSMCs as compared to those without thrombosis. Using machine learning techniques, these parameters sufficiently segregated patients who subsequently developed post-angioplasty and AVF thrombosis, both of which are examples of post vascular injury-induced thrombosis. Furthermore, administration of Kyn to a mouse model of vascular injury increased thrombosis, which was subsequently attenuated by an AHR inhibitor [89]. This series of experiments delineated CKD-specific mechanisms driving thrombosis and also uncovered therapeutic targets that can be used for further development of safer antithrombotic agents for CKD-associated thrombosis. Taken together, all these studies strongly suggest that indolic metabolites and Kyn activate AHR signaling to regulate TF, the primary trigger of extrinsic coagulation. This event culminates in thrombus formation at the site of reactive vascular bed, created after vascular injury (rupture of atherosclerotic plaque or intervention [86,87,88,89].

## 4. Effect of Uremic Toxins on Cardiomyocytes

CKD patients often develop congestive heart failure (both preserved and reduced ejection fraction), and their illness is often pathologically characterized by cardiac fibrosis and remodeling [6]. In a prospective study of 433 ESRD patients, 74% were reported to develop ventricular hypertrophy [92]. Several molecular mechanisms have been proposed to account for effects of UT on the heart. IS was found to increase ROS levels within cardiomyocytes and inhibited AMP-activated protein kinase (AMPK). These events resulted in increased protein synthesis and cell volume of cultured neonatal rat cardiomyocytes, consistent with the cardiac hypertrophy observed in CKD patients [93]. Similar findings including IS-induced heart fibrosis were reported in rats treated with IS [94]. In another set of studies, IS affected endoplasmic reticulum stress modulators and induced apoptosis in the cardiomyocyte cell line H9C2 [95]. pCS administered to mice induced apoptosis in cardiomyocytes, resulting in diastolic dysfunction, partially attributed to augmented ROS [96]. pCS also reduced spontaneous contraction of cultured cardiomyocytes, attributed to a damaging effect on cellular gap junctions [97]. While cardiomyopathy is common in CKD patients and is the driver of frequent hospitalization and mortality in these patients, little is known about how these UTs induce these pathological changes.

## 5. The Effects of Uremic Toxins on Monocytes/Macrophages, Polymorphonuclear Cells and Platelets

### 5.1. The Influence of Uremic Toxins on Macrophages and Monocytes

Macrophages are key contributors to vascular inflammation and progression of atherosclerosis, and the uremic milieu has been shown to enhance their activation and pro-inflammatory effect [98,99,100,101,102]. Nakano et al. showed that IS is transported into macrophages by organic anion transporter (OAT) P2B1 [103]. Once inside the cell, IS increases the expression of ubiquitin-specific peptidase 5, leading to inhibition of the ubiquitin–proteasome pathway, and consequent accumulation of δ-like ligand 4 (DLL4). DLL4 is responsible for the cleavage of the intracellular domain of Notch1, a member of the Notch transmembrane receptor family that has been implicated in proinflammatory activation of macrophages and development of CVD. Augmented levels of DLL4 enhance cleavage of the intracellular domain of Notch1, which undergoes nuclear translocation to serve as a transcriptional regulator and induce proinflammatory genes. In 5/6 nephrectomy atherosclerotic mice, DLL4 and Notch1 intracellular domain expression were found to be increased in macrophages residing the atherosclerotic plaque, supporting an in vivo activation of Notch signaling in CKD milieu. Treatment with an anti-DLL4 antibody targeted to macrophages through lipid coated nanoparticles inhibited production of inflammatory cytokines, decreased macrophage accumulation, and decreased progression of atherosclerosis in CKD mice. Similarly, mice receiving 4 weeks of intraperitoneal IS and subsequently silenced for OAT P2B1 and DLL4 in macrophages showed decreased vascular inflammation and reduced atherosclerotic plaque burden as compared to controls [103]. This interesting study showed the role of IS and uremic milieu in manipulating macrophages in the atherosclerotic plaque in a CKD milieu. In a separate study, THP-1-derived macrophages exposed to IS in vitro were shown to have increased production of inflammatory cytokines such as TNF-α and IL-1β. Notably, these macrophages were shown to have significantly decreased activity of ATP-binding cassette transporter A1 (ABCA1), a lipid transporter essential for cholesterol efflux, thus potentially leading to accelerated development of foam cells and progression of atherosclerosis [104].

Monocytes express elevated levels of angiotensin converting enzyme (ACE) in ESRD patients, which has been hypothesized to contribute to accelerated atherosclerosis [101,105]. In an in vitro study, both human primary monocytes and THP-1 cells treated with uremic serum showed higher ACE expression. The authors surmised that this ACE overexpression might promote M2 macrophage differentiation, with associated pro-inflammatory and pro-atherosclerotic properties. The monocytes with higher ACE2 expressions showed increased levels of inflammatory cytokines TNF-α and IL-6, as well as increased levels of arginase-1 (Arg1), which is a marker of M2 cells present in plaques, and may itself also promote plaque stabilization and proliferation of vSMCs. ACE overexpressing monocytes were also shown to have greater expression of MCP-1 and its ligand CCR2, potentially leading to enhanced transmigration through the endothelium. Additionally, ACE overexpression in such monocytes led to upregulation of adhesion molecules such as ICAM-1 and VCAM-1, molecules considered essential to the initial steps of atherosclerotic plaque formation [101,102,105]. One of the hallmarks of CVD secondary to kidney disease is intravascular macrophage inflammation. An in vitro study showed that IS activated AHR/NFκB/MAPK cascades in macrophages, causing an inflammatory response [106]. A study involving 268 HD patients followed for 36 months showed that a high neutrophil-to-lymphocyte ratio was an independent predictor of cardiovascular mortality [107]. Another uremic toxic, phenylacetic acid (PAA) [108], increases oxidative burst and inflammatory cytokine production in polymorphonuclear cells, which could contribute to augmented cardiovascular risk [109]. Collectively, these studies demonstrated UTs inducing substantial alterations in monocytes and macrophages, associated with vascular inflammation and cardiovascular pathology.

### 5.2. Effect of Uremic Toxins on Platelets

The CKD milieu profoundly alters platelet functions, which contributes to atherothrombosis in these patients [38]. Indeed, earlier studies showed that renal dysfunction is associated with inappropriate platelet activation [110,111]. Mouse platelets treated with IS displayed increased activity in response to collagen and thrombin [112]. IS also augmented platelet-derived microparticles and platelet-monocyte aggregates, which are also contributors to vascular dysfunction [112]. In accordance, IS enhanced thrombus formation as measured in a flow chamber assay and carotid artery thrombosis model that improved with AST-120 administration (an oral adsorbent of uremic toxins) or klotho [112]. Interestingly, ROS induction of p38 MAPK signaling in platelets is a driver in IS-mediated platelet activation, both in vivo and in vitro [112].

## 6. Uremic Toxin-Induced Microparticles and Cardiovascular Disease

Microparticles (MPs) are fragments derived from cell membranes, and contain various membrane-associated receptors and ligands [113]. MPs can originate from endothelial cells, platelets, erythrocytes, polymorphonuclear cells, monocytes, and lymphocytes, and bear cell-type-specific markers. On the one hand, MPs may help facilitate intracellular communications and maintenance of homeostasis, but on the other, they might trigger a deleterious pro-inflammatory response [114,115,116,117]. MPs bind to and enter target cells by various endocytic pathways, and inside the cell may activate inflammatory pathways, thereby causing functional impairment [116,118]. MPs are released secondary to plasma membrane remodeling in the setting of programmed cell death, injury, or cellular activation [117].

Endothelial injury secondary to UTs induces release of endothelial microparticles (EMPs), often being specifically mediated by IS, pCS, IAA, and inorganic phosphate (accumulating secondary to hyperphosphatemia) [119,120,121,122,123]. pCS promotes EMP formation through an effect on Rho kinase and resultant cytoskeletal reorganization [75,124]. Likewise, IS induces the release of EMPs from endothelial cells in a dose-dependent manner via upregulation of the p38 MAPK signaling pathway [125]. Erythrocytes treated with IS or IAA showed increased phosphatidylserine (PS) exposure along with release of PS-laden MPs, in turn leading to a hypercoagulable state [126].

MPs from different origins can regulate distinct phenotypes in the vascular system. For example, a study analyzed plasma from ESRD patients and found an increase in MPs of platelet, erythrocyte, and endothelial origin [127]. However, only EMPs correlated with loss of flow-mediated relaxation, whereas MPs of other origins did not. The same study also showed that in vitro, only EMPs impaired endothelial cGMP generation and NO release, leading to a compromised endothelium-dependent relaxation [127]. Furthermore, EMPs isolated from CKD patients have been shown to induce osteocalcin expression in EPCs, vSMCs, and fibroblasts, leading to vascular calcification [128]. Concordantly, the number of circulating EMPs is greater in CKD patients with vascular calcification than without it, and the increase in EMPs in CKD patients is associated with a decrease in the number of EPCs, suggesting a role for EMPs in impaired tissue renewal [128]. EMPs can also regulate immune cells, which in turn can influence inflammation and thrombosis [129].

IS-induced EMPs (IsEMPs) in particular have been shown to contain miRNAs that reduce the angiogenic capacity of EPCs, as well as upregulate expression of NF-κB and p53 in them, all of which culminate in an inflammatory response and EPC apoptosis [130]. Accordingly, in vitro studies have shown that ECs treated with TNF-α produced EMPs that can increase monocytic TF-dependent procoagulant activity [131]. A separate study explored the role of IsEMPs in NH, one of the main causes of vascular access malfunction in patients on hemodialysis. It showed that vessels exposed to EMPs ex vivo developed significantly greater NH and vSMC proliferation via heightened activation of the TGF-β signaling pathway [121]. In accordance with this study, EMPs collected from the culture media of human umbilical vascular endothelial cells treated with IS augmented NH and vSMC proliferation in cultured porcine internal jugular veins, concomitant with activation of the MAPK pathway, and in a similar manner to TGF-β [121].

IS-induced EMP release can be modulated by pharmacological approaches. Ryu et al. demonstrated that IS induced generation of EMPs in culture media of cells [132]. Such EMPs stimulated vSMC proliferation in a concentration-dependent manner, upregulated TGF-β, and promoted the TGF-β signaling pathway, as illustrated by phosphorylation of downstream targets, including Akt, ERK1/2, p38 MAPK, and Smad3, all of which being significantly inhibited by an anti-TGF-β antibody and inhibitors of ERK and Smad. Several compounds have been investigated to suppress IS-induced EMP production, including losartan, lovastatin, clopidogrel, and mesoglycan [125]. All of these drugs inhibited EMP generation induced by IS at concentrations between 10–50 μM. However, the suppressive effect was highest with clopidogrel, which significantly suppressed MAPK signaling activated by IS in endothelial cells.

## 7. Conclusions

It has become clear that pathology in one organ can induce systemic effects resulting in pathology in other organs. The case of CKD-associated accumulation of UTs and their untoward effects on different components of the cardiovascular system is an example of this paradigm. While epidemiological studies pointed to CKD as an independent risk factor for various forms of cardiovascular disease, the molecular mechanisms by which this occurs have only recently been gradually elucidated. To this end, the current review provides a comprehensive overview of cellular signaling pathways triggered by a set of well-established cardiovascular uremic toxins. As illustrated in Figure 1, different types of UTs have the ability to derange molecular pathways in various cell types, whether vSMCs, ECs, platelets, or cardiomyocytes, all of which are central to a healthy cardiovascular system. Understanding which biological processes are impacted in these cells could lead to more targeted treatments for different forms of CVD in CKD.
